# Extremely Coherent Microwave Emission from Spin Torque Oscillator Stabilized by Phase Locked Loop

**DOI:** 10.1038/srep18134

**Published:** 2015-12-11

**Authors:** Shingo Tamaru, Hitoshi Kubota, Kay Yakushiji, Shinji Yuasa, Akio Fukushima

**Affiliations:** 1 Spintronics Research Center, National Institute of Advanced Industrial Science and Technology (AIST), Tsukuba, 305-8568, Japan

## Abstract

Spin torque oscillator (STO) has been attracting a great deal of attention as a candidate for the next generation microwave signal sources for various modern electronics systems since its advent. However, the phase noise of STOs under free running oscillation is still too large to be used in practical microwave applications, thus an industrially viable means to stabilize its oscillation has been strongly sought. Here we demonstrate implementation of a phase locked loop using a STO as a voltage controlled oscillator (VCO) that generates a 7.344 GHz microwave signal stabilized by a 153 MHz reference signal. Spectrum measurement showed successful phase locking of the microwave signal to the reference signal, characterized by an extremely narrow oscillation peak with a linewidth of less than the measurement limit of 1 Hz. This demonstration should be a major breakthrough toward various practical applications of STOs.

Flexible yet stable frequency synthesis of microwave signals is a key enabler for various modern electronics systems including wireless communication, radar, global positioning system to name a few. Spin torque oscillators (STOs), which generate a continuous microwave signal by first exciting spin precession through spin transfer torque (STT)^1^,^2^ and next converting it to an electrical signal through magneto-resistance (MR) effect, possess many attractive features for the above mentioned applications, such as nano-scale dimension due to self-oscillation without resonance circuits, low power operation, wide frequency tunability and compatibility with semiconductor fabrication processes. These make STO a promising candidate for the next generation microwave signal sources, and therefore since its initial experimental demonstrations^3^,^4^,^5^,^6^, extensive research efforts have been devoted to improve the STO’s basic performances such as emission power and quality factor (Q factor)^7^,^8^,^9^,^10^,^11^,^12^,^13^,^14^,^15^,^16^,^17^,^18^,^19^,^20^,^21^,^22^,^23^,^24^,^25^,^26^,^27^,^28^,^29^. Deac *et al.* demonstrated a large emission power (>0.1 μW) in MTJ-based STO^13^ due to the giant MR ratio of MgO-based magnetic tunnel junction (MTJ)^30^,^31^. To date, emission powers of MTJ-based STOs have reached up to a few μW^22^,^25^. Moreover, the use of highly coherent out-of-plane precession of free layer magnetization was found to be effective for reducing the spectral linewidth and thus enhancing the Q factor of a free-running STO^18^,^21^,^23^. Such out-of-plane precession can be realized by inducing a perpendicular magnetic anisotropy to the free layer and/or applying a perpendicular magnetic field.

In spite of these extensive efforts, however, there is still a profound gap between the current phase noise level of free running STOs and the industry requirements, which have been hampering adoption of STOs in real electronics systems^27^,^28^. Therefore, some kind of industrially viable means to stabilize the STO oscillation has been strongly sought to overcome this difficulty. Techniques many researchers have been pursuing so far for this purpose are mutual phase locking between two STOs mediated by spin waves^8^,^9^,^11^,^14^, and so-called injection locking^7^,^12^,^19^,^20^,^29^. In the injection locking, an external radio frequency (RF) signal (either RF current^7^,^12^,^20^ or RF magnetic field^19^,^29^) of the same frequency as the STO oscillation or its fractional multiple is injected into the STO to stabilize the oscillation. It has been demonstrated that this technique can actually synchronize the STO oscillation to the injected RF signal, resulting in basically the same spectral purity as the injected RF signal, thus effectively suppressing the phase noise of the STO oscillation. However, this technique has found little practical use thus far because it requires another signal source generating the same or even higher frequency of the STO oscillation. On the other hand, the most widely used technique to stabilize the oscillation of conventional microwave oscillators is phase locked loop (PLL)^33^. In this technique, the phase of the RF signal generated by a voltage controlled oscillator (VCO), whose frequency can be fine tuned by an applied voltage, is compared with that of a reference signal. The phase error between these two signals is converted into a voltage signal and fed back to the VCO control input to dynamically tune the frequency such that the phase error is always minimized within the bandwidth of the feedback loop. The most notable advantage of PLL over the conventional injection locking is that the frequency of the reference signal, *f*_ref_, is usually much lower than that of the VCO output signal. This allows the use of a reference signal in the 10–100 MHz frequency range, which can be readily generated by a highly stable signal source such as temperature compensated crystal oscillator (TCXO) or Rubidium oscillator, to stabilize a much higher frequency signal generated by the VCO. PLL has been so commonly used to stabilize various microwave oscillators that it is quite natural to try to stabilize the STO oscillation by PLL as well^16^. Such an attempt has actually been made by Keller *et al.* which demonstrated successful phase locking of the output signal from a vortex STO to a reference signal^17^. However, although their work is definitely the first major milestone for generating a highly coherent STO output signal, the low frequency due to the use of a vortex STO and the fact that the reference frequency is the same as the STO frequency in their work implied that there still exist many technological difficulties in order for STOs to generate a GHz range output signal with a sufficiently high coherence for practical microwave applications.

Here we report on the first demonstration of STO based PLL working in the microwave frequency range, which generates an extremely coherent 7.344 GHz microwave signal phase locked to a 153 MHz reference signal. Spectrum measurement showed a linewidth less than the measurement limit (1 Hz) under phase locked oscillation. The keys for our success were fabrication of high performance STOs showing a sufficiently high Q factor as well as a PLL circuit customized to deal with a wide phase noise spectrum of the STO.

## Experimental

We fabricated STOs consisting of a nano-pillar-type MgO-based MTJ with nearly perpendicularly magnetized FeB free layer and in-plane magnetized CoFeB reference layer. With the help of perpendicular magnetic anisotropy (PMA) of the FeB free layer, the free layer was perpendicularly magnetized by an out-of-plane applied field that is much smaller than the saturation magnetization of the free layer, which resulted in out-of-plane precession. The structure and fabrication processes of the MTJ-based STO used in this work are described in Methods.

**Figure 1:**
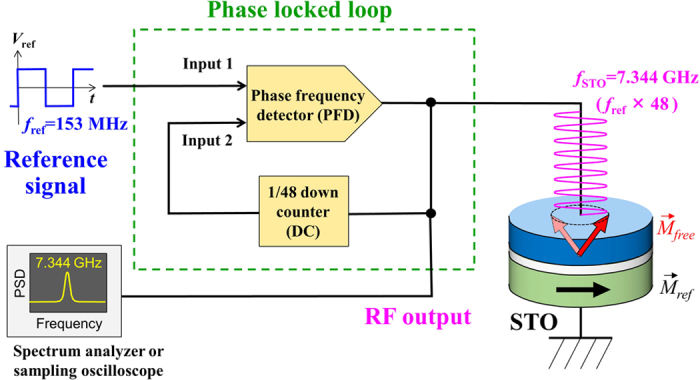
Simplified block diagram of the phase locked loop built in this work. The functionality of each block is as follows. Spin torque oscillator (STO) is nominally biased to oscillate at around 7.344 GHz. Down counter (DC) down counts the input signal by 48, thus converting 7.344 GHz RF signal to 153 MHz. Phase frequency detector (PFD) generates a voltage signal proportional to the phase error between the two input signals, which is fed back to the STO to dynamically stabilize the oscillation such that the phase error is always minimized.

Figure 1 shows the simplified block diagram of the PLL built in this work (a more detailed block diagram is presented in Methods). The STO was nominally biased to oscillate at around 7.344 GHz. The STO output signal was first down counted by 48 by a down counter (DC) and sent to one input of the phase frequency detector (PFD). A 153 MHz reference signal was sent to the other input of the PFD, and the PFD generated a voltage signal proportional to the phase error between the two input signals (phase error signal), *V*_PES_. *V*_PES_ was low-pass filtered by a loop filter (not shown in Fig. 1, see Methods) and eventually fed back to the STO to dynamically tune the oscillation frequency such that the phase error is always minimized. The STO output signal was also sent to either a spectrum analyzer for frequency domain spectrum measurement or sampling oscilloscope for time domain waveform measurement.

## Results and Discussion

**Figure 2:**
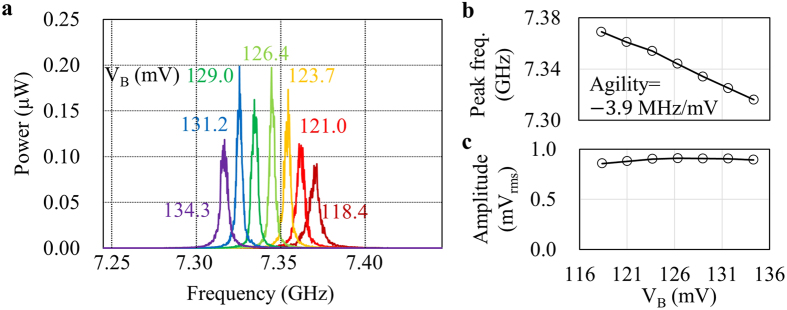
Free running characteristics of the STO used in this work. (**a**) Spectra of the RF signal generated by the STO under different bias voltages. These spectra are observed by a spectrum analyzer with a frequency span of 200 MHz and resolution bandwidth of 1 MHz. (**b**) Peak frequency and (**c**) amplitude as a function of bias voltage. Agility is the slope of the frequency around the nominal bias point of *V*_B_ = 126.4 mV.

Figure 2 summarizes the free running characteristics of the STO used in this work. Figure 2a shows the spectra of the STO output signal under different bias voltages, *V*_B_. The oscillation peak shifts to a lower frequency as *V*_B_ is increased from 118.4 mV up to 134.3 mV. The frequency span, *f*_span_, of these measurements is set to 200 MHz, and it has been confirmed that no other peaks are observed outside of this frequency span. Figure 2b plots the peak frequency as a function of *V*_B_. This STO showed a monotonic red shift in this bias voltage range with an agility coefficient of –3.9 MHz/mV around the center of this plot^32^. Figure 2c plots the amplitude of the STO output signal as a function of *V*_B_. In general, it is desirable for a VCO to generate a constant amplitude over the frequency tuning range for PLL applications. If not only the frequency but also the amplitude of the VCO output is affected by the control voltage, changes in *V*_PES_ are directly converted to an amplitude fluctuation that cannot be stabilized by PLL, thus degrading the spectral purity when the VCO output frequency is tuned by PLL. This plot shows that the amplitude of the STO output varies only ±3.5% over the *V*_B_ range used for frequency tuning. These results prove that this STO shows a good performance as a VCO.

**Figure 3:**
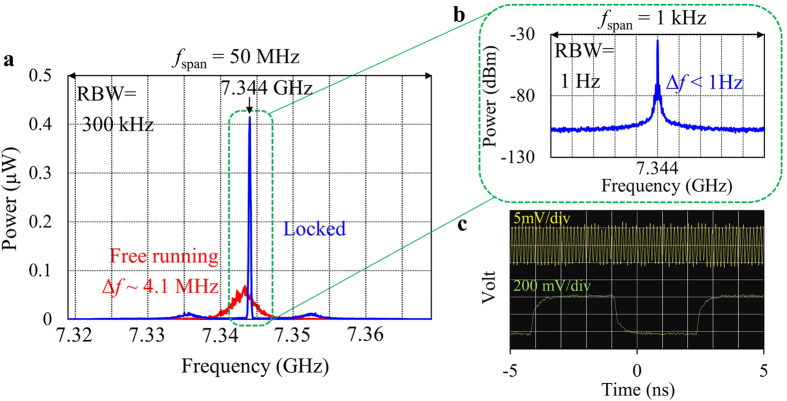
(**a**) Spectra of the STO output under two operation conditions. Red and blue curves are for free running and phase locked oscillations, respectively. These spectra are observed by a spectrum analyzer with a frequency span of 50 MHz and resolution bandwidth of 300 kHz. (**b**) Magnified spectrum of the phase locked STO output signal around 7.344 GHz with a frequency span of 1 kHz and resolution bandwidth of 1 Hz. (**c**) Time domain waveform observed by a sampling oscilloscope with a bandwidth of 20 GHz and averaging of 64. Yellow and green lines show the STO output signal and the reference signal, respectively.

Figure 3 demonstrates the performance of the STO based PLL built in this work. Figure 3a shows the comparison of two STO output spectra. The red and blue curves are for free running and phase locked oscillations, respectively. In this plot, *f*_span_ and resolution bandwidth (RBW) are set to 50 MHz and 300 kHz, respectively. The red curve shows a linewidth of about 4.1 MHz, corresponding to a Q factor of about 1800. This is quite high for nano-pillar-type STOs but still not high enough for most practical applications. On the other hand, the blue curve shows a very sharp peak right at 7.344 GHz. Figure 3b is a magnified view of this peak with *f*_span_ of 1 kHz and RBW of 1 Hz. An extremely narrow peak still appears right at 7.344 GHz. Notice that Fig. 3a is a linear power plot while Fig. 3b is a logarithmic plot because the noise floor is too low to be displayed in a linear plot when RBW is set to 1 Hz. The linewidth, defined as the difference between lower and higher frequency points that give 3 dB lower power than the peak, is definitely less than the measurement limit of 1 Hz. This is a clear signature that the PLL successfully phase locked the STO output signal to the reference signal. Figure 3c shows the time domain waveform of the STO output signal as yellow, together with the 153 MHz reference signal as green, respectively. This waveform acquisition was triggered by the reference signal, and the fact that a sine wave was observed as the STO output signal on a sampling oscilloscope means that the STO output and the reference signal have timing correlation. This is another clear signature that the STO output signal was successfully phase locked to the reference signal.

**Figure 4:**
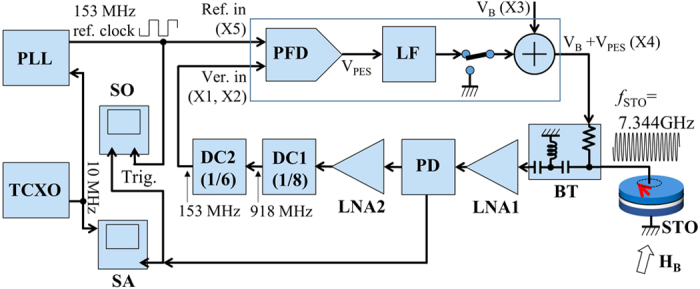
Detailed block diagram of the PLL built in this work. Each functional block is labeled as follows. STO: Spin torque oscillator, BT: Bias-Tee, LNA1: 1st Low Noise Amplifier, PD: Power Divider, LNA2: 2nd Low Noise Amplifier, DC1: 1st Down Counter (fixed to 8), DC2: 2nd Down Counter (programmed to 6), PFD: Phase Frequency Detector, LF: Loop Filter, SA: Spectrum Analyzer, SO: Sampling Oscilloscope, TCXO: Temperature compensated crystal oscillator generating 10 MHz master clock, PLL: PLL synthesizer generating 153 MHz reference signal. The detailed explanation about the functionality of each block and operation principle are given in Method. The labels X1–X5 are the connector ID shown in the circuit diagram (Figure s1) in Supplementary information.

The following two factors contributed to the successful phase locking of the STO output signal demonstrated in this work. One is the use of a high performance STO having a narrow linewidth in the free running state. In general, a PLL circuit has to have a loop bandwidth sufficiently wider than the linewidth of the VCO in order to suppress phase noise of it. The narrow linewidth of the STO used in this work accordingly lowered the required bandwidth of the feedback loop down to a practically achievable level. The other is a feedback loop customized for STO. The loop bandwidth of several MHz, needed to suppress phase noise of the STO with a linewidth of 4.1 MHz, is still two to three orders of magnitude wider than normal PLL circuits designed to stabilize an ordinary VCO. This inevitably necessitates some special attentions to be paid in the circuit design. The feedback loop was designed to have as wide bandwidth as possible by using high speed operational amplifiers for the PFD and LF blocks, and adjusting the open loop gain to be as large as possible within the stability limit. In addition, the STO is a single port device, in which the two signals, the bias voltage (=*V*_B_ + *V*_PES_) input and RF signal output, share a single electrode (and the other electrode is grounded). In reality, the output of the LF block contains both the real V_PES_ (distributed in the low frequency range, mostly below 8 MHz) and spurious signals at the reference frequency and its higher harmonics (at 153 MHz and its integer multiples in this work). These signals, particularly the spurious signals, are harmful for the PLL operation if they leak into the LNA1 input, as it can make the output waveform noisier, or cause even loss of phase locking due to miscount in the down counter. For this reason, a high-pass (HP) filter with a cut-off frequency of about 1 GHz was inserted to the RF path of the bias-tee to completely block the bias voltage from leaking into the RF path while the RF signal generated by the STO can pass through to the LNA1 input (see Fig. 4 in Method for details).

Closer observations of the plots in Fig. 3 give more information about the performance of the STO based PLL. In Fig. 3a, the blue line shows two small broad peaks at around 8 MHz offset from the center frequency, and the power level of the blue curve is higher than the red curve outside of these broad peaks. These results indicate the following. First, the loop bandwidth of the PLL circuit is around 8 MHz. Second, the feedback loop is somewhat underdamped, resulting in some noise boost near the edge of the loop bandwidth, which should be caused by the highest possible open loop gain within the stability limit as mentioned above. The noise increase outside of the two broad peaks at 8 MHz offset is probably due to additional noise introduced by the high speed operational amplifiers and logic gates composing either DC or PFD blocks. From the time domain waveform shown in Fig. 3c, the total timing jitter is estimated to be about 20 ps(rms). These performance indicators (phase noise and timing jitter) are still about two orders of magnitude worse than current state-of-the-art PLL circuits commercially available, meaning that further performance improvement is needed for STO based PLLs to be actually used in practical microwave applications. It is inferred that the larger phase noise (or timing jitter in time domain) of the STO based PLL than commercial PLL circuits is caused by the following two factors. One is that the spectral linewidth of the STO under free running oscillation is several orders of magnitude wider than that of typical semiconductor based VCOs. We measured the spectrum of one commercial VCO generating about 3.8 GHz under free running operation for comparison purpose, and found that its linewidth is less than 100 Hz, although it slowly moved around without any stabilization. This indicates that the STO shows a much larger phase noise than typical VCOs. The other is that the loop bandwidth of 8 MHz is not wide enough to suppress phase noise over sufficiently wide frequency range. Based on the above considerations, there are three guidelines to further reduce the phase noise and timing jitter. First is to adopt a STO having an even higher Q factor under free running oscillation^23^,^25^. A higher Q factor means that less phase noise is introduced by the STO, thus obviously should lead to a smaller phase noise when the STO output is phase locked. Second is to design the loop bandwidth even wider and critically damped. For this direction, the entire circuit has to be miniaturized to reduce the total propagation delay and each component has to be fast enough. Third is to reduce noise introduced by the PLL circuit itself, which may be attained by optimizing the PLL circuit topology and parameters. Because the present work is our first demonstration of PLL-stabilized STO, there should still be a large room for further improvements.

In conclusion, we developed a PLL using a STO as a VCO that generates a 7.344 GHz microwave signal stabilized by a 153 MHz reference signal. The PLL showed successful phase locking of the STO output signal to the reference signal. The spectrum observation of the phase locked STO output signal showed an extremely narrow linewidth of much less than the measurement limit of 1 Hz. This successful demonstration of STO based PLL is considered to be a major breakthrough toward various practical microwave applications such as wireless communication, radar, and global positioning system.

## Methods

### STO fabrication

The MTJ film for STO was deposited on a thermally oxidized Si substrate by sputtering, followed by annealing at 360 °C for one hour under in-plane magnetic field of 1 T.^21^ The stack structure of the MTJ film was PtMn (15)/CoFe (2.5)/Ru (0.85)/CoFeB (3)/MgO (1)/FeB (2)/MgO (1)/Ta (5) (from bottom to top, thickness in nm). STOs were then fabricated by patterning the MTJ film into a nano-pillar with various diameters ranging from 100 nm up to 400 nm using e-beam lithography, ion beam etching, *etc* (for more details about the sample fabrication process, see ref. 21). These STO’s were tested and the one showing the best performance as a VCO (high Q factor and sufficiently wide tuning range), which was of 400 nm in diameter on the wafer tested, was chosen for the PLL experiment. The chosen STO’s MR ratio and resistance for parallel magnetic state were 47% and 11 Ω, respectively. As stated in Experimental, application of an externa field is needed to saturate the free layer magnetization along the out-of-plane direction, because the PMA of this MTJ is not strong enough. The typical RH curve and the magnetic configuration of the STO fabricated on the same wafer under each bias field are shown in Fig. 2 of Ref. 34. It should be noted that the MR ratio was not close to the highest because the resistance-area (RA) product was ultra-low (only 1.4 Ωm^2^). The medium MR ratio and ultra-low RA product were important for the high-performance STO operation.

### PLL circuit

Figure 4 shows a detailed block diagram of the PLL circuit built in this work. The nominal bias voltage, *V*_B_, is applied to the STO through the analog adder and the DC path of the bias-tee (BT) to excite oscillation at around 7.344 GHz. The RF signal generated by the STO goes through the RF path of the BT, and sent to the low noise amplifier 1 (LNA1). The BT is custom built to have a high-pass filter with a cut-off frequency of about 1.1 GHz in order to prevent *V*_PES_ signal from leaking from the DC path to the RF path. The half of the RF signal coming out of the power divider (PD) is further amplified by the low noise amplifier 2 (LNA2) to be compatible with the digital input of the down counter 1 (DC1). It is first down counted by 8 (fixed) in DC1, next by 6 (programmed) in DC2 (48 in total), thus converted down to 153 MHz, and sent to one input of the phase frequency detector (PFD). The 153 MHz reference signal is sent to the other input of the PFD, and the PFD generates a voltage signal proportional to the phase error between these two signals (*V*_PES_). The *V*_PES_ signal is low pass filtered by the loop filter (LF), added to *V*_B_ and eventually fed back to the STO to dynamically tune the frequency such that the phase error is always minimized. The other half of the PD output is sent to either the spectrum analyzer or sampling oscilloscope. The 153 MHz reference signal is generated by a commercial PLL circuit referenced to a 10 MHz master clock generated by a TCXO. This 10 MHz master clock is also sent to the reference input of the spectrum analyzer. The use of a common 10 MHz master clock in the PLL generating the 153 MHz reference signal and the spectrum analyzer means that these blocks share the same frequency reference, thus completely eliminating frequency shift in the spectrum measurement. The 153 MHz reference signal is also sent to the sampling oscilloscope to trigger wave acquisition.

### Measurement procedure

The STO was mounted on a sample stage capable of applying a constant bias magnetic field along arbitrary directions. Initially, the bias field angle and magnitude were optimized to give the narrowest linewidth and reasonably large output signal by applying a constant bias voltage across the STO while scanning the bias field direction and magnitude. This was repeated by changing the bias voltage to find out the best biasing condition. For the particular STO used in the PLL experiment, the narrowest oscillation peak was obtained under a bias voltage of 126.4 mV and a bias field of 3320 Oe applied along 19 degrees away from the film normal toward the direction such that the FeB free layer magnetization was tilted parallel to the CoFeB reference layer magnetization. Next, the bias voltage was swept over a sufficiently wide voltage range while the spectrum was observed to make sure that the peak shows a smooth and monotonic red shift and therefore the STO can be used as a VCO. This is necessary because if the oscillation peak shows non-monotonic frequency shift or sudden change in its peak shape or position, the feedback loop may be trapped at such irregular transitions. Lastly, the feedback loop of the PLL circuit was closed to dynamically stabilize the STO oscillation, and its output was observed by either a spectrum analyzer or sampling oscilloscope.

## Additional Information

**How to cite this article**: Tamaru, S. *et al.* Extremely Coherent Microwave Emission from Spin Torque Oscillator Stabilized by Phase Locked Loop. *Sci. Rep.* **5**, 18134; doi: 10.1038/srep18134 (2015).

## Supplementary Material

Supplementary Information

## References

[b1] Slonczewski J. C. Current-driven excitation of magnetic multilayers. J. Magn. Magn. Mater. 159, L1–L7 (1996).

[b2] Berger L. Emission of spin waves by a magnetic multilayer traversed by a current. Phys. Rev. B 54, 9353–9358 (1996). 10.1103/physrevb.54.9353 9984672

[b3] Kiselev S. I. *et al.* Microwave oscillations of a nanomagnet driven by a spinpolarized current. Nature 425, 380 (2003). 14508483 10.1038/nature01967

[b4] Rippard W. H., Pufall M. R., Kaka S., Russek S. E. & Silva T. J. Direct-current induced dynamics in Co90Fe10/Ni80Fe20 point contacts. Phys. Rev. Lett. 92, 27201 (2004). 10.1103/PhysRevLett.92.027201 14753964

[b5] Krivorotov I. N. *et al.* Time-domain measurements of nanomagnet dynamics driven by spin-transfer torques. Science 307, 228–231 (2005). 15653496 10.1126/science.1105722

[b6] Rippard W. H., Pufall M. R., Kaka S., Silva T. J. & Russek S. E. Current-driven microwave dynamics in magnetic point contacts as a function of applied field angle. Phys. Rev. B 70, 100406 (2004).

[b7] Rippard W. H., Pufall M. R., Kaka S., Silva T. J. & Russek S. E. Injection locking and phase control of spin transfer oscillators. Phys. Rev. Lett. 95, 067203 (2005). 16090984 10.1103/PhysRevLett.95.067203

[b8] Kaka S. *et al.* Mutual phase-locking of microwave spin torque nano-oscillators. Nature 437, 389–392 (2005). 16163351 10.1038/nature04035

[b9] Mancoff F. B., Rizzo N. D., Engel B. N. & Tehrani S. Phase-locking in double-point-contact spin-transfer devices. Nature 437, 393–395 (2005). 16163352 10.1038/nature04036

[b10] Mistral Q. *et al.* Current-driven microwave oscillations in current perpendicular-to-plane spin-valve nanopillars. Appl. Phys. Lett. 88, 192507 (2006).

[b11] Pufall M. R. *et al.* Electrical measurement of spin-wave interactions of proximate spin transfer nanooscillators. Phys. Rev. Lett. 97, 087206 (2006). 17026331 10.1103/PhysRevLett.97.087206

[b12] Georges B. *et al.* Coupling Efficiency for Phase Locking of a Spin Transfer Nano-Oscillator to a Microwave Current. Phys. Rev. Lett. 101, 017201 (2008). 18764148 10.1103/PhysRevLett.101.017201

[b13] Deac A. M. *et al.* Bias-driven high-power microwave emission from MgO-based tunnel magnetoresistance devices. Nature Physics 4, 803 (2008).

[b14] Ruotolo A. *et al.* Phase-locking of magnetic vortices mediated by antivortices. Nature Nanotechnology 4, 528–532 (2009). 10.1038/nnano.2009.143 19662017

[b15] Bonetti S., Muduli P., Mancoff F. & Åkerman J. Spin torque oscillator frequency versus magnetic field angle: The prospect of operation beyond 65 GHz. Appl. Phys. Lett. 94, 102507 (2009).

[b16] Villard P. *et al.* A GHz Spintronic-Based RF Oscillator. IEEE J. Solid-State Circuits, 45 214 (2010).

[b17] Keller M. W., Kos A. B., Silva T. J., Rippard W. H. & Pufall M. R. Time domain measurement of phase noise in a spin torque oscillator. Appl. Phys. Lett. 94 193105 (2009).

[b18] Rippard W. H. *et al.* Spin-transfer dynamics in spin valves with out-of-plane magnetized CoNi free layers, Phys. Rev. B 81, 014426 (2010).

[b19] Urazhdin S., Tabor P., Tiberkevich V. & Slavin A. Fractional Synchronization of Spin-Torque Nano-Oscillators. Phys. Rev. Lett. 105, 104101 (2010). 20867522 10.1103/PhysRevLett.105.104101

[b20] Dussaux A. *et al.* Phase locking of vortex based spin transfer oscillators to a microwave current. Appl. Phys. Lett. 98 132506 (2011).

[b21] Kubota H. *et al.* Spin-Torque Oscillator Based on Magnetic Tunnel Junction with a Perpendicularly Magnetized Free Layer and In-Plane Magnetized Polarizer. Appl. Phys. Express 6, 103003 (2013).

[b22] Maehara H. *et al.* Large Emission Power over 2 μW with High Q Factor Obtained from Nanocontact MagneticTunnel-Junction-Based Spin Torque Oscillator. Appl. Phys. Express 6, 113005 (2013).

[b23] Maehara H. *et al.* High Q factor over 3000 due to out-of-plane precession in nano-contact spin-torque oscillator based on magnetic tunnel junctions. Appl. Phys. Express 7, 023003 (2014).

[b24] Tamaru S. *et al.* Bias field angle dependence of the self-oscillation of spin torque oscillators having a perpendicularly magnetized free layer and in-plane magnetized reference layer. Appl. Phys. Express 7, 063005 (2014).

[b25] Tsunegi S. *et al.* High emission power and Q factor in spin torque vortex oscillator consisting of FeB free layer. Appl. Phys. Express 7, 063009 (2014).

[b26] Nagasawa T., Kudo K., Suto S., Mizushima K. & Sato R. Large-amplitude, narrow-linewidth microwave emission in a dual free-layer MgO spin-torque oscillator. Appl. Phys Lett. 105, 182406 (2014).

[b27] Choi Hyun Seok *et al.* Spin nano-oscillator-based wireless communication. Sci. Rep. 4, 5486 (2014). 24976064 10.1038/srep05486 PMC4074786

[b28] Chen T. *et al.* Integration of GMR-based spin torque oscillators and CMOS circuitry. Solid-State Electron. 111, 91 (2015).

[b29] Hamadeh A. *et al.* Perfect and robust phase-locking of a spin transfer vortex nano-oscillator to an external microwave source. Appl. Phys. Lett. 104, 022408 (2014).

[b30] Parkin S. S. P. *et al.* Giant tunnelling magnetoresistance at room temperature with MgO (100) tunnel barriers. Nature Mater. 3, 862 (2004). 15516928 10.1038/nmat1256

[b31] Yuasa S. *et al.* Giant room-temperature magnetoresistance in single-crystal Fe/MgO/Fe magnetic tunnel junctions. Nature Mater. 3.868 (2004). 15516927 10.1038/nmat1257

[b32] Taniguchi T. *et al.* Critical Field of Spin Torque Oscillator with Perpendicularly Magnetized Free Layer. Appl. Phys. Express 6, 123003 (2013).

[b33] Gardner F. M. Phaselock Techniques. John Wiley & Sons, 2005.

[b34] Tamaru S. *et al.* Bias field angle dependence of the self-oscillation of spin torque oscillators having a perpendicularly magnetized free layer and in-plane magnetized reference layer. Appl. Phys. Express 7, 063005 (2014).

