# Supplementary material for: Extremely Coherent Microwave Emission from Spin Torque Oscillator Stabilized by Phase Locked Loop
Source: Sci Rep. 2015 Dec 11;5:18134. doi: 10.1038/srep18134 (PMC4676054; doi:10.1038/srep18134)
Supplement: Supplementary Information [file srep18134-s1.pdf]

Supplementary Information

Circuit design guideline of the STO based PLL

Shingo Tamaru, Hitoshi Kubota*, Kay Yakushiji, Shinji Yuasa and Akio Fukushima

Spintronics Research Center, National Institute of Advanced Industrial Science and Technology (AIST), Tsukuba, 305-8568, Japan

*Email: hit-kubota@aist.go.jp

PLL circuit design

This supplementary information presents the design information of the STO based PLL test bench built in this work.

Table s1 summarizes the microwave components used in the STO based PLL. The gain and insertion loss values are for 7.344 GHz. The typical signal amplitudes and frequencies in the microwave path are as follows. The raw output signal of the STO used in this work is about 4.5 mV(rms), which corresponds to 0.4 μW. This is first attenuated by the HP filter of the BT and then amplified by the LNA1, becoming about 360 mV(rms) at the output of the LNA1. This signal is again attenuated by the PD down to about 170 mV(rms). This is larger than the maximum input level of the LNA2, so the signal waveform at the output of the LNA2 is distorted (clipped with some asymmetry) with an amplitude of about 1.2 V(pp). This signal is first sent to the input of the DC1 and down counted by a factor of 8, becoming 918 MHz, then is further down counted by a factor of 6 in the DC2, eventually becoming 153 MHz before being sent to the PFD circuit.

Figure s1 shows the circuit diagram of the PFD, LF and analog adder blocks (see Figure s1 for the signal labels of U3). The 153 MHz reference signal input into the connector X5 was first sent to an ECL buffer (U1:MC100EP16DG) and then to the R input of the PFD (U3: MC100EP140DG). The differential output signal of the DC2 input into the connectors X1 and X2, which is a STO output signal down counted by 48, was also first sent to an ECL buffer (U2: MC100EP16DG) and then to the FB input of the PFD. The PFD generated a pulse signal, whose width is proportional to the phase difference between the R and FB signals, only to either $\mathrm{U}/\overline{\mathrm{U}}$ or $\mathrm{D}/\overline{\mathrm{D}}$ terminals depending on whether the phase of the FB signal either leads to or lags behind the R signal. These output signals were sent to an analog subtractor composed of a high speed operational amplifier (U4: THS4271D) to be bundled into a single line, V_{PES}. This was

fed to a loop filter circuit composed of a high speed operational amplifier with output amplitude limiter (U5: OPA699ID). The loop filter was a standard lag/lead type, whose frequency response was set by R7 (39 Ω), R31 (430 Ω) and C26 (470 pF). The amplitude of the U5 output was limited to ±200 mV in order to prevent V_{PES} from going beyond the tuning range of V_B of the STO (the maximum V_{PES} swing was ±200 mV at the U5 output, ±176 mV at the U6 output and ±18.5 mV at the STO, which is nearly the same as the total tuning range of V_B as shown in Fig. 2b). The nominal value of V_B was set by an external voltage source programmed by a host computer, input into the connector X3 and sent to a differential instrumentation amplifier (U7: AMP03GPZ). V_B and V_{PES} were added by an analog adder composed of another high speed operational amplifier (U6: THS4031CD). This output signal was output from the connector X3, and injected into the STO through a 200 Ω resister embedded in the BT block. Thus the signal was attenuated by a factor of 11 Ω / (200 Ω + 11 Ω) = 0.052 at the STO.

Figure s2 shows a photo of the entire PLL test bench. The STO wafer was put under a high frequency probe. The bias magnetic field was generated by a permanent magnet sitting beneath the STO wafer. In the real experiment, the STO performance was first measured as a function of biasing condition, such as magnitude of V_B, magnitude and direction of H_B, by a probe station capable of applying a magnetic field along arbitrary directions. The STO showing the best performance on the probe station was contacted by the probe on the PLL test bench, and the magnetic field was fine adjusted by moving the permanent magnet to obtain the same performance as observed on the probe station. The microwave signal generated by the STO propagated through a coax cable with a length of about 30 cm connecting the probe and the BT, passing through the RF path of the BT and was eventually fed to the LNA1. The LNA1 output was split into two paths at the PD, and one of them was further amplified by the LNA2 and down counted by the DC1 and DC2. This signal was sent to the PFD and LF circuit board explained above, and the $V_B + V_{PES}$ was fed back to the STO through the DC path of the BT. The total signal propagation path length was approximately 1.2 m in the current setup.

Table and Figure legends

Table s1. Microwave components used in the PLL circuit, and the key parameter for each. The gain and insertion loss values are measured at 7.344 GHz.

Figure s1. Circuit diagram of the PFD and LF blocks of the PLL circuit.

Figure s2. Photo of the entire PLL test bench.

Block in Fig. 4	Manufacturer and model	Key performance parameters
BT	Custom	Insertion loss: 2.8 dB<br>f_c: 1.1 GHz
LNA1	B&Z Technology<br>BZ-00101600-221040-252020	Gain: 41 dB<br>Bandwidth: 16 GHz
PD	Mini-Circuits ZFRSC-183+	Insertion loss: 6.3 dB
LNA2	Anritsu AH14149A	Gain: 27 dB<br>Bandwidth: 10 GHz
DC1	Picosecond pulse labs Model 5650	Max frequency: 16 GHz<br>Division ratio: 8 (fixed)
DC2	On Semiconductor MC100EP016	Max frequency: >1 GHz<br>Division ratio: 6 (programmed)

Table s1 Kubota et al.

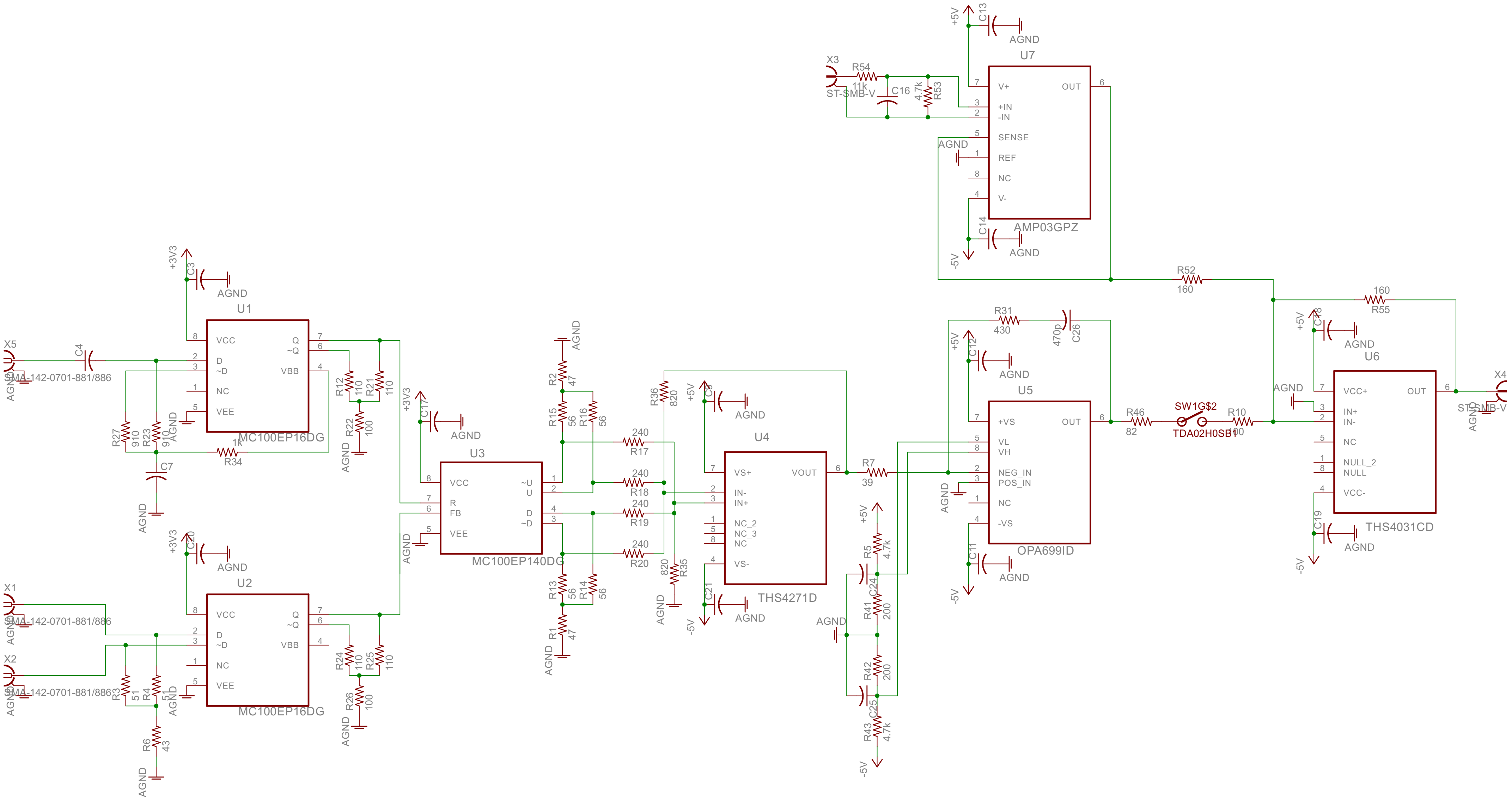

X3
R54
11k
ST-SMB-V
C16
4.7k
R53
+5V
C13
AGND
U7
V+
+IN
-IN
SENSE
REF
NC
V-
OUT
AMP03GPZ
C14
-5V
R52
160
R55
160
+3V3
C3
U1
VCC
D
~D
NC
VEE
Q
~Q
VBB
MC100EP16DG
X5
C4
SMA-142-0701-881/886
R27
910
R23
910
1k
R34
C7
R12
110
R21
110
R22
100
U2
MC100EP16DG
C20
X1
X2
R3
51
R4
51
R6
43
R24
110
R25
110
R26
100
C17
U3
VCC
R
FB
VEE
~U
U
D
~D
MC100EP140DG
R2
47
R15
56
R16
56
R13
56
R14
56
R1
47
240
R17
240
R18
240
R19
240
R20
R36
820
820
R35
C9
U4
VS+
IN-
IN+
NC_2
NC_3
NC
VS-
VOUT
THS4271D
C21
R7
39
R5
4.7k
C24
R41
200
R42
200
C25
R43
4.7k
R31
430
470p
C26
C12
U5
+VS
VL
VH
NEG_IN
POS_IN
NC
-VS
OUT
OPA699ID
C11
R46
82
SW1G$2
TDA02H0SB1
R10
100
C18
U6
VCC+
IN+
IN-
NC
NULL_2
NULL
VCC-
OUT
THS4031CD
C19
X4
ST-SMB-V

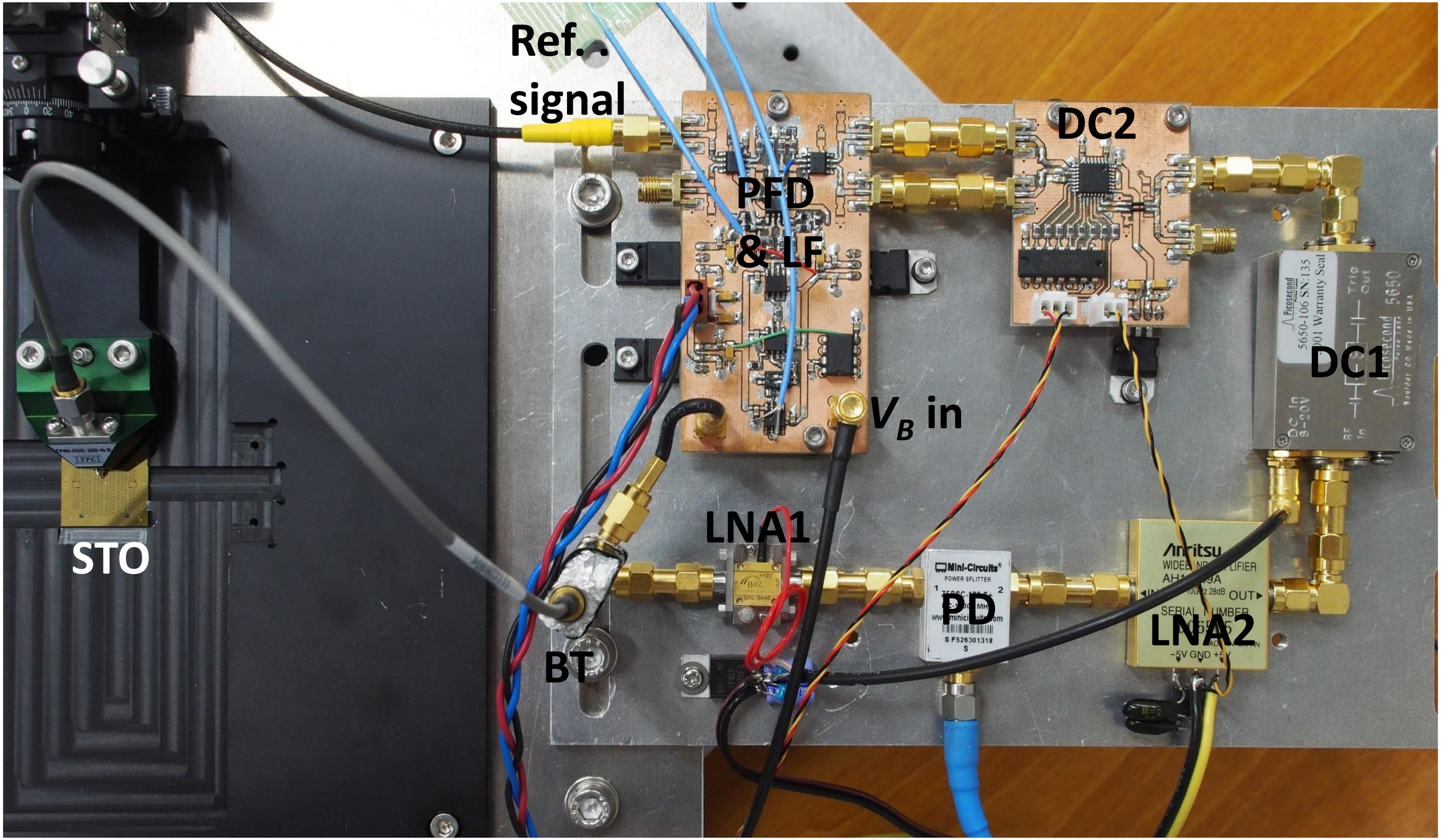

Figure s2 Kubota et al.
